# Functional Neurological Disorder as a Disorder of Sensorimotor Inference Under Stress: A Neurocomputational Account

**DOI:** 10.1016/j.bpsgos.2026.100825

**Published:** 2026-09-09

**Authors:** Andrea Putica

**Affiliations:** Melbourne School of Psychological Sciences, Parkville, Victoria, Australia

**Keywords:** Functional neurological disorder, Interoception, Precision weighting, Predictive processing, Sensorimotor, Stress neurobiology

## Abstract

Functional neurological disorder (FND) sits at the intersection of psychiatry and neurology, causing disabling motor and sensory symptoms despite intact peripheral pathways. FND may reflect altered precision weighting within neural systems, such that the brain generates the dysfunction it predicts. Stress is among the most consistent precipitants; however, how it produces functional symptoms remains mechanistically unspecified. The present account proposes that FND arises from the overprecision of maladaptive priors relative to afferent sensory evidence within insula–cingulate-sensorimotor circuits under stress-mediated neuromodulation. Chronic or traumatic stress is proposed to recalibrate precision through hypothalamic-pituitary-adrenal axis dysregulation and noradrenergic modulation. The proposed result is that overly precise expectations about bodily dysfunction dominate proprioceptive and motor inference and override ascending feedback. What may distinguish FND from other stress-linked conditions is the sensorimotor locus of these priors and their implementation in motor control systems. The mechanism is mapped onto circuits including the anterior insula, dorsal anterior cingulate cortex, supplementary motor area, and temporoparietal junction. Falsifiable predictions are derived at algorithmic, neural, and physiological levels, each with an explicit disconfirmation criterion. A discriminative prediction separates this account from competing models. Attenuation of insula prediction-error signaling during voluntary movement should be greater with stress-system dysregulation and may ease under acute reduction of arousal, whereas sensory-gain and attentional-capture models predict no such modulation. The framework offers a possible account of why verbal reassurance alone may be insufficient and provides a computational basis for physiotherapy and, if validated, for pretreatment precision profiling to help guide treatment planning.

## Plain Language Summary

Functional neurological disorder causes real, disabling symptoms despite normal brain scans, and stress is a well-known trigger; however, brain models of the symptoms and the neurobiology of stress have remained separate. This conceptual review unites them, showing how stress hormones recalibrate the brain’s predictions about the body. The result of this integration is a distinctive, testable prediction that separates the proposed mechanism from competing explanations.

Functional neurological disorder (FND) causes disabling motor and sensory symptoms and substantial impairment despite the absence of structural pathology; however, its generative mechanism remains poorly specified (1, 2, 3, 4). Health care costs rival those of structural neurological disease (3), and outcomes are often persistent or relapse after treatment (5,6). A mechanistic account capable of guiding treatment selection and identifying computational targets is lacking.

Stress is one of the most consistent precipitants of FND (7), with meta-analytic evidence indicating that stressful life events and maltreatment are reported in 44% to 78% of FND samples, at odds ratios substantially exceeding those reported for comparable neurological conditions (7,8). Physical triggers, including injury, infection, and surgery, are also common across subtypes (9). The term “stress” is used here in the allostatic sense, referring to anticipated perturbation of physiological regulation that increases energetic demand (10). This definition is not restricted to negatively valenced or consciously experienced stressors and does not assume a single underlying process. Sustained perturbation engaging noradrenergic and glucocorticoid signaling is the component proposed to recalibrate precision. Psychodynamic, dissociative, and attentional accounts lack the computational specificity to generate falsifiable predictions or guide targeted intervention (11,12). Predictive processing theory provides a formal framework in which perception and action emerge from hierarchical Bayesian inference (13,14), and stress neurobiology documents the neuroendocrine pathways through which stress alters the neural systems in which this inference is thought to be implemented (15, 16, 17). The relationship between these 2 levels of explanation remains mechanistically underspecified.

Although candidate modulators of precision have been proposed, existing accounts have not connected an identifiable antecedent to precision miscalibration within a falsifiable, circuit-specific pathway. The Bayesian account of functional symptoms locates abnormally precise priors in the synaptic gain of superficial pyramidal cells under dopaminergic and cholinergic modulation, shaped by sensory salience arising from injury or illness, affective arousal, and sociocultural illness expectations (18). More recent work models precision as a dynamic quantity that surges with arousal, settles toward baseline, and recalibrates over longer timescales, situating this within an affective-interoceptive-proprioceptive-spinal hierarchy (19). At the neurophysiological level, the amplitude of the heartbeat-evoked potential (HEP) is proposed to index interoceptive precision and changes before functional seizures in a way that tracks semiology. Motile seizures are preceded by reduced amplitude, a pattern that has been interpreted as being consistent with attenuated precision on cardiac interoceptive prediction errors and a low-precision, dissociated bodily state. Nonmotile seizures are preceded by increased amplitude (20). Reduced HEP amplitude preceding motile seizures is consistent with the present account because lower afferent precision would increase the relative influence of standing priors. By contrast, increased HEP amplitude before nonmotile seizures is consistent with greater precision of ascending prediction errors rather than of priors, placing this presentation outside the account’s current scope (20). These findings suggest that seizure semiology may track the direction of precision imbalance. Prior accounts have also proposed injury, illness, affective arousal, and autonomic arousal as potential antecedents of precision change (18,19). Therefore, the present framework focuses more narrowly on specifying a testable pathway from allostatic perturbation to sensorimotor precision.

More specifically, chronic and traumatic stress, operating via hypothalamic-pituitary-adrenal (HPA) axis dysregulation and noradrenergic modulation, are proposed to recalibrate precision weighting in the circuits that generate bodily experience. This mechanism is mapped onto directional predictions about circuit-level connectivity testable with existing neuroimaging methods, and explicit falsification criteria are derived at algorithmic, neural, and physiological levels, including a discriminative prediction that distinguishes the account from competing sensory-gain and attentional-capture models.

## Predictive Processing and Interoceptive Inference

Under predictive processing, the brain maintains a generative model of expected sensory input and updates it by minimizing the mismatch between predicted and received signals (13,14,21). Precision weighting determines whether priors or prediction errors dominate inference. High prior precision discounts sensory evidence, whereas high error precision forces belief revision (22). Miscalibrated precision therefore biases inference in either direction.

Interoception refers to the sense of the body’s internal physiological condition, conveyed by visceral, humoral, thermal, and related afferents and represented centrally in the insular cortex (23,24). It is conventionally distinguished from proprioception, which signals the position and movement of the body, and from exteroception. Interoception operates within this architecture as a hierarchical inference across cortical and subcortical circuits (25,26). The anterior insula, dorsal anterior cingulate cortex (dACC), and brainstem visceral relay nuclei implement a generative model of the body (27, 28, 29, 30), in which priors about expected internal states are compared with ascending signals, and mismatches update bodily awareness, autonomic regulation, and motor planning. An interoceptive prior is an expectation about a bodily state, for example, the expectation that cardiac sensations signal threat; limb weakness is a proprioceptive expectation. An interoceptive hyperprior is a higher-order expectation not about the state itself but about the precision that lower levels should afford to interoceptive priors and their associated prediction error (31). One possible computational formalization places the relevant imbalance at the hyperprior level, as an aberrant expectation that ascending interoceptive prediction errors are unreliable and should be downweighted relative to the prior. The proposed downstream consequence is a first-order prior about bodily dysfunction that comes to dominate inference, not because the bodily state is genuinely as predicted but because the precision balance set by the hyperprior prevents ascending evidence from correcting it. In functional dizziness, for example, the account would predict that a prior of instability persists despite vestibular and proprioceptive signals indicating stability because the governing hyperprior assigns insufficient precision to those ascending signals to revise the prior.

The account proposes that interoceptive, proprioceptive, and sensorimotor signals remain anatomically distinct but are integrated under common precision control at higher hierarchical levels. Within this formulation, the hyperprior would govern the precision assigned to first-order priors across the relevant channels, namely, proprioceptive and motor priors of movement failure or loss of agency, and exteroceptive priors of sensory abnormality, with interoceptive priors implicated where symptoms are visceral or affective (Table 1). By assigning these priors disproportionately high precision relative to ascending evidence, the hyperprior allows them to dominate inference. The framework therefore proposes that prediction errors signaling intact motor function are suppressed, and descending predictions shape motor output, so that the dysfunction predicted becomes the dysfunction produced. This rigidity reflects attenuated belief updating rather than irreversibility, since persistence depends on prior learning strength, the availability of corrective signals, and the current neuromodulatory state.

**Table 1:** Mapping Stress-Calibrated Overprecise Priors Onto FND Symptom Domains

FND Symptom Domain	Symptom-Related Prior	Hierarchical Locus	Primary Circuit	Testable Prediction
Motor (Weakness, Tremor, Gait)	Anticipated movement failure or loss of voluntary control	Motor planning and execution	Anterior insula→SMA and premotor cortex; reduced PFC–sensorimotor coupling	Attenuated proprioceptive prediction-error encoding during voluntary movement despite intact peripheral signaling
Sensory (Anesthesia, Visual)	Expected sensory loss or abnormality	Sensory cortical inference	Insula–sensory cortex connectivity; reduced thalamocortical precision	Reduced somatosensory mismatch negativity with preserved brainstem evoked potentials
Seizure-Like (PNES)	Anticipated loss of bodily control under arousal, with disembodiment in motile presentations	Interoceptive-autonomic-motor integration	Amygdala-insula–SMA coupling; limbic–motor overflow; altered interoceptive precision in insula	Direction of HEP change preceding events tracks semiology: reduced HEP amplitude precedes motile seizures (prior-dominant inference); increased HEP amplitude precedes nonmotile seizures (precise ascending errors, outside the present scope) (20)
Dissociative (Depersonalization, Agency Disturbance)	Expected disconnection of self from body or from action	Self-model representations at higher levels of abstraction	Reduced anterior insula–TPJ coupling; disrupted agency network	Sustained reduction in interoceptive prediction-error signaling with reduced TPJ integration, dissociable from time-locked HEP changes preceding functional seizures

The table maps symptom domains onto the framework’s proposed mechanisms; the nonmotile functional seizure pattern is included as a boundary condition rather than as confirmation of a prior-dominant inference.

FND, functional neurological disorder; HEP, heartbeat-evoked potential; PFC, prefrontal cortex; PNES, psychogenic nonepileptic seizures; SMA, supplementary motor area; TPJ, temporoparietal junction.

Precision imbalance may manifest as functional neurological symptoms when overprecise dysfunction priors are instantiated within sensorimotor systems that directly govern action execution, rather than within affective or belief-based hierarchies. Specificity may therefore lie in the locus of miscalibration, not a distinct precision pathology. The recalibration mechanism is proposed to be shared with other stress-linked conditions (depression, anxiety, and posttraumatic stress disorder [PTSD]), and the cited evidence is drawn from samples in which such comorbidities are common. The framework predicts that patients with FND and psychiatric control participants with comparable stress exposure share a general precision miscalibration, while differing in the channel and circuit locus at which it is expressed.

## How Stress May Recalibrate Precision Weighting

Within the present account, chronic stress is proposed to shape priors through repeated exposure to bodily states linked to threat or uncontrollable arousal (25,32); over time, these states become embedded as high-precision priors that bias inference even in safe contexts. The following subsections specify how this recalibration operates at the neuroendocrine, receptor, and network levels.

### Neuroendocrine Calibration

The HPA axis is one candidate pathway through which stress may alter precision weighting. Chronic and traumatic stress can disrupt normal diurnal cortisol variation and impair recovery (33, 34, 35), with downstream effects on limbic and prefrontal systems (15,36). Reduced circadian glucocorticoid variation and altered receptor occupancy may therefore bias the amygdala, insula, and medial prefrontal cortex toward anticipatory threat. Whether such changes are accompanied by reduced error correction or altered precision weighting has not been established. Stress-related changes in prefrontal and salience systems are unlikely to be uniform. Reduced prefrontal engagement and arousal-related amplification of already-salient signals have both been observed under stress (15,36,37), but neither determines whether priors or prediction errors dominate inference. In the present account, prior dominance emerges when latent illness-related priors provide the relevant content and attention is directed toward the body; under these conditions, reduced prefrontal engagement is hypothesized to limit correction of those priors. The resulting prediction is a relative downweighting of ascending error signals within salience and sensorimotor circuits.

HPA findings in FND are heterogeneous, including elevated baseline cortisol (38), flattened awakening responses (39), and associations between cortisol amplitude and clinical outcome (40). Allostatic-load research distinguishes acute hypercortisolism from hypocortisolism and flattened slopes associated with chronic or traumatic exposure (41,42). In chronic FND samples, the framework predicts blunted diurnal slope and flattened awakening response, with cortisol amplitude associated with symptom chronicity. Elevated tonic baseline with preserved slope in a chronic sample would be inconsistent with this prediction. Illness duration is therefore expected to moderate this profile, with hypocortisolism emerging more clearly in longer-standing FND. Heterogeneity across existing studies likely reflects variation in illness duration, chronicity of stress exposure, and measurement protocol. Because stress-related HPA changes can persist beyond the period of exposure (43), it is possible, though not established, that such persistence contributes to the chronicity of FND symptoms (5). The cross-sectional nature of current data and the longitudinal designs required to test this are discussed in Supplementary 1a.

### Receptor-Level Mechanisms

The neuroendocrine and receptor-level changes described here provide candidate biological routes through which stress could influence precision weighting, although their convergence on prior-dominant inference has not been demonstrated. Cortisol acts through high-affinity mineralocorticoid receptors (MRs) and low-affinity glucocorticoid receptors (GRs). These are coexpressed in limbic regions and mediate complementary, time- and dose-dependent actions rather than separate or opposing ones (44); chronic stress is proposed to disturb this MR/GR balance. Norepinephrine provides a parallel route. Prefrontal α_2A_-adrenergic receptors support stable top-down control of prediction (15,45,46), whereas arousal-driven noradrenergic signaling in the amygdala and insula increases the gain on salient, high-priority signals while suppressing competing low-priority inputs (37,47,48). Chronic stress is proposed to downregulate prefrontal α_2A_ control and sensitize arousal-driven selectivity. These mechanisms alter gain rather than content; which representations are prioritized is attributed here to latent illness priors and bodily attention. As noted above, specificity arises not from the endocrine disturbance, which is transdiagnostic, but from the sensorimotor locus at which precision is recalibrated (see Supplementary 1a for a full receptor-level elaboration).

### Network-Level Reallocation

At the systems level, acute and chronic stress are associated with heightened activity in salience-network nodes, including the anterior insula and dACC, alongside reduced engagement of prefrontal regions that support cognitive flexibility (15,49,50). These regions are better described as nodes within overlapping systems than as modules: a default mode/allostatic system supporting anticipatory regulation, a salience or cingulo-opercular action mode system arbitrating between predictions and ascending evidence, a central autonomic network, and skeletomotor systems. The recalibration proposed here is a shift in relative weighting across these motifs rather than a change localized to one region. Regional language is retained below for correspondence with the FND imaging literature, which is reported at that level. The salience network is part of broader precision-weighting processes rather than a separate mechanism. Activity within these networks is proposed to influence the precision afforded to interoceptive and bodily signals and the arbitration between internally generated predictions and ascending evidence. For example, the anterior insula is thought to weight how strongly an ascending bodily signal counts against a standing prediction, whereas the dACC is proposed to track its reliability (27, 28, 29, 30); when these nodes assign low precision to ascending evidence, predictions go uncorrected. Neuroimaging findings in FND are broadly consistent with this pattern (4). Stress effects on these systems are nonlinear and time and context dependent, and the present account does not claim that stress maps onto a single fixed global configuration. Within salience and sensorimotor circuits, however, the direction of the effect is not arbitrary. Within the present framework, these asymmetric receptor-level mechanisms are hypothesized to contribute to greater relative weighting of entrenched priors over corrective evidence under chronic exposures associated with FND. The framework’s discriminative predictions (Table 2) test this specific directional effect, not a uniform global shift. PTSD comparisons and extended salience network elaboration are presented in Supplementary 1b.

**Table 2:** Stress-Specific Falsifiable Predictions of the Stress-Calibrated Account of FND

Level of Analysis	Prediction	Method	Disconfirmation Criterion
Algorithmic	Within FND, individually estimated prior-to-sensory precision ratios should covary with cumulative stress exposure, such that patients with greater stress exposure (early-life adversity, recent stressful events, and chronic stress burden) show higher prior-to-sensory precision ratios. The relationship should be specific to precision parameters and not reduced to learning rate or response-bias parameters.	Cardiac interoception task with parametrically varied temporal jitter and probabilistic contextual cues, fitted with hierarchical Bayesian observer models (69) (see Supplementary 4). Within-FND correlation of precision parameters with validated cumulative stress measures (e.g., Adverse Childhood Experiences Inventory, Life Events Scale, and Perceived Stress Scale).	If precision ratios within FND do not covary with stress exposure, or if any covariation is fully accounted for by learning-rate or response-bias parameters rather than precision parameters, the stress-calibration mechanism is not supported.
Neural Implementation	Within FND, the magnitude of insula–dACC resting-state connectivity should covary with concurrent neuroendocrine indices of stress (HPA function, autonomic balance), and experimentally induced acute stress should produce a transient increase in this connectivity that is not seen in matched control participants. Treatment-related reductions in connectivity should be moderated by changes in HPA function rather than being independent of them.	Resting-state fMRI with concurrent salivary cortisol sampling; an additional acute-stress challenge scan (e.g., Trier Social Stress Test variant) in a subset of patients and matched control participants; pre-post treatment scans with paired HPA assessment.	If insula–dACC connectivity within FND is independent of neuroendocrine indices, if acute stress does not differentially modulate this connectivity in FND vs. control groups, or if treatment-related connectivity changes occur independently of HPA changes, the stress-calibration mechanism is not supported.
Discriminative	The magnitude of attenuation in insula prediction-error signaling during voluntary movement should be greater in patients with FND with greater stress-system dysregulation and should be acutely modulated by experimentally manipulated arousal (e.g., pharmacological β-adrenergic blockade should partially restore insula prediction-error responses). Competing accounts predict no such stress- or arousal-dependent modulation: pure sensory-gain accounts predict stable attenuation independent of stress state, and attentional-capture accounts predict modulation by attentional manipulation rather than by arousal manipulation.	Event-related fMRI during motor tasks with proprioceptive perturbation, comparing insula BOLD response across patients with FND stratified by stress-system indices; pharmacological challenge with propranolol vs. placebo in a within-participant design. Adjustment for heart-rate change and self-reported anxiety; an attentional manipulation matched for subjective arousal as an active comparison, to establish that any effect is not reproduced by attentional redirection alone.	If insula prediction-error attenuation in FND is independent of stress-system indices and is not modulated by arousal manipulation, the stress-calibration mechanism is not supported (even if the general attenuation finding holds, which would support a stress-independent precision-imbalance account but not the present stress-calibrated account). The discriminative test additionally requires that the BOLD attenuation pattern be accompanied by a reduced sensory-precision parameter in the algorithmic-row computational fit and by a behavioral signature of reduced influence of late-arriving sensory evidence; if BOLD attenuation occurs without these convergent signatures, the result is consistent with elevated attentional precision on a competing channel rather than with reduced sensory precision on the perturbation channel, and the discriminative claim is not supported. Propranolol alters heart rate, peripheral autonomic feedback, subjective arousal, attentional state, and symptom-related anxiety in addition to any effect on precision weighting. A positive result under β-blockade is therefore consistent with, but not diagnostic of, arousal-dependent precision recalibration. Absent the convergent signatures specified above, this row is a weaker test than the algorithmic and neural rows and should not be treated as decisive.
Predictive (Noncircular)	Baseline computational precision parameters (prior-to-sensory precision ratio from the algorithmic-row task) should predict subsequent treatment response in a prospective cohort, with higher prior-to-sensory precision ratios at baseline predicting poorer response to interventions that depend on prediction-error generation (e.g., physiotherapy protocols using distraction or dual-task paradigms). The prediction should hold after adjustment for baseline symptom severity, comorbid anxiety and depression, and demographic variables.	Prospective cohort design in which the algorithmic-row task is administered at intake, prior to a standardized treatment protocol, with treatment response measured at a prespecified follow-up using a validated FND outcome measure. Hierarchical regression of treatment response on baseline precision parameters, adjusting for symptom severity, comorbidity, and demographics.	If baseline precision parameters do not predict treatment response after adjustment, or if the relationship is fully accounted for by baseline symptom severity rather than by precision parameters specifically, the framework lacks predictive validity beyond contemporaneous association, and the circularity concern is not resolved.
Physiological	Within FND, flattened cortisol diurnal slopes and reduced heart rate variability should correlate with computational precision parameters (prior-to-sensory precision ratio) and with symptom chronicity. Patients with greater neuroendocrine dysregulation should show higher prior precision on the algorithmic task. The relationship should be specific to FND and not present in matched anxious or depressed comparison samples with comparable HPA dysregulation.	Salivary cortisol sampling (waking, +30 minutes, afternoon, and evening) and ambulatory HRV recording in the same FND cohort completing the algorithmic-level task, with correlation of neuroendocrine indices to individually estimated precision parameters; an anxious-depressed comparison sample matched for HPA dysregulation.	If neuroendocrine markers do not correlate with precision parameters within FND, or if the relationship is equally present in comparison samples with comparable HPA dysregulation, the stress-calibration mechanism (and its specificity to FND) lacks empirical support. Elevated tonic baseline cortisol with preserved diurnal slope in a chronic FND sample would also be inconsistent with the predicted chronic-stress hypocortisolism pattern, independent of the within-FND covariations specified above.

Each prediction tests a within-FND, arousal-dependent, or prospective relationship that is specifically entailed by the stress-calibration mechanism and would not be predicted by precision-imbalance accounts lacking a stress component. Predictions consistent with the general precision-imbalance account of FND (e.g., FND vs. control differences in prior-to-sensory precision ratios and insula–dACC connectivity) are presented in Supplementary 4 as replication targets rather than as discriminative tests of the present account. The discriminative claim of the present account depends on convergence between the algorithmic-row computational-model parameter pattern and the discriminative-row neural and arousal-dependence patterns; failure of an individual row would weaken the corresponding mechanistic claim, whereas failure of the predicted cross-modal convergence would count more strongly against the integrated account. The predictive row provides a noncircular test in which baseline precision parameters are required to predict treatment response after adjustment for baseline symptom severity, addressing the risk that precision parameters fitted in symptomatic patients reflect downstream consequences of the symptoms themselves.

BOLD, blood oxygen level–dependent; dACC, dorsal anterior cingulate cortex; fMRI, functional magnetic resonance imaging; FND, functional neurological disorder; HPA, hypothalamic-pituitary-adrenal; HRV, heart rate variability; SMA, supplementary motor area.

## From Stress-Calibrated Priors to FND Symptoms

The preceding sections describe a stress-driven precision miscalibration mechanism that could affect any inferential domain; what determines whether the outcome is FND rather than another stress-linked condition is the locus at which overprecise priors become entrenched. In sensorimotor systems, motor control depends on continuous precision-weighted prediction-error correction, so disrupted precision is proposed to alter motor output through descending predictions to spinal motor neurons, producing functional deficits via the insula–supplementary motor area (SMA) pathway. Precision miscalibration in threat-detection circuits would instead be expected to produce hypervigilance, as proposed in PTSD, whereas miscalibration in interoceptive-affective circuits may contribute to persistent distress, as in generalized anxiety. Neither directly disrupts voluntary movement. The critical question is therefore what localizes stress-driven miscalibration to sensorimotor rather than affective or threat-detection systems.

Three candidate factors may determine the sensorimotor locus of prior entrenchment, each developed with extended evidence and testable predictions in Supplementary 2. They operate at different timescales and are proposed to contribute distinct components to channel selection. Latent illness–related priors supply the content of the relevant representations. The functional integration of limbic, salience, and motor circuits supplies the architectural bias toward sensorimotor over affective channels. Neuroendocrine variation supplies the gain, determining how much precision a given stressor reallocates. The present framework therefore predicts that localization to sensorimotor channels depends on the joint contribution of all 3 factors, with no single factor sufficient, and that their interaction should predict FND more strongly than their independent effects.

Once entrenched, dysfunction priors operate below conscious volitional control. The precision-weighted prior is proposed to override proprioceptive feedback, rendering corrective prediction errors computationally inert (18,51). Hoover’s sign, in which involuntary hip extension is preserved during contralateral hip flexion despite apparent voluntary weakness (11), is consistent with the present account and with related predictive-coding interpretations. Preserved involuntary movement indicates intact motor capacity, whereas the present account proposes that altered precision weighting prevents proprioceptive evidence from overriding the dysfunction prior during voluntary action. The sign is illustrative rather than uniquely diagnostic of the precision framework.

Overprecise dysfunction priors are proposed to be self-maintaining through several feedback loops. On this account, dysfunction predictions would generate descending autonomic and motor commands producing the predicted bodily state (increased muscle tension, altered gait, and autonomic changes), which would then be registered as confirming evidence, further increasing prior precision. Avoidance reduces exposure to corrective evidence, and investigations that fail to yield a structural diagnosis may paradoxically reinforce dysfunction expectations by heightening attention to bodily signals (5). Together, these processes may further stabilize the dysfunction prior over time.

Precision allocation is treated here as dynamic rather than fixed, which offers a possible account of the variability, context sensitivity, and suggestibility characteristic of FND. Distraction can attenuate functional motor symptoms (11); within the present framework, this is interpreted as a temporary reduction in the precision of the dysfunction prior, allowing ascending evidence greater influence. Suggestion and attention are proposed to alter the relative weighting of competing priors through related processes. Sudden symptom onset (5) may reflect nonlinear precision dynamics, in which gradual increases in prior precision produce an abrupt shift once a threshold is crossed.

The specific symptom profile is proposed to depend on 2 features: the functional channel in which the overprecise priors are expressed and their level of abstraction within that channel. On the proposed mapping, priors in motor-planning regions would produce weakness, priors in somatosensory cortices would produce anesthesia-like deficits, and priors at higher levels of abstraction, where self-model representations are computed, would produce depersonalization and disturbances of agency. A complementary interoceptive contribution operates within the same precision framework rather than as a separate route, governed by the same hyperprior structure and most relevant where symptoms have prominent visceral or autonomic features (see Supplementary 1c). Table 1 maps each symptom domain to its prior content, hierarchical locus, primary circuit, and testable prediction.

## Neural Implementation

The proposed mechanism is mapped onto interactions among the anterior insula, dACC, SMA, prefrontal cortex, and temporoparietal junction (TPJ) within the broader networks described above (4,52). Within this account, these regions are assigned candidate roles in body-related prediction-error processing, precision modulation, motor implementation, corrective control, and agency-related integration, respectively. These assignments are provisional interpretations of the imaging literature rather than directly demonstrated computational functions (see Supplementary 3).

The anterior insula shows elevated functional connectivity with the SMA and limbic structures in FND (4,53), alongside altered limbic responses and limbic–motor interactions during processing of emotional stimuli (47,54). In healthy individuals, the anterior insula integrates afferent information with contextual predictions to generate a coherent body-state representation (27). In FND, this integration is proposed to be skewed at the intermediate levels of the cortical hierarchy at which bodily inference is computed, where the body-related dysfunction prior emerges in the context of a precision surge, consistent with the Bayesian account of functional symptoms (18). Higher levels, where abstract beliefs about agency, illness identity, and self-model are represented, contribute via top-down precision allocation rather than by carrying the dysfunction prediction directly. This would yield a body-state representation dominated by predicted rather than incoming physiological information.

The dACC has been implicated in precision modulation under uncertainty (28,29); altered dACC engagement during affective processing (12), ACC hypometabolism at rest in functional seizures (55), and altered prefrontal–sensorimotor coupling (53,56) are consistent with a reduced capacity to correct dysfunction priors downstream. The SMA sits at the convergence of descending intention signals and modulatory input from salience and interoceptive circuits. Within the proposed model, when overprecise dysfunction priors are entrenched at intermediate sensorimotor and interoceptive levels, the SMA implements motor output consistent with those priors, producing motor suppression or aberrant motor programs. The contribution of the anterior insula and salience network is precision modulation, allocating precision to interoceptive prediction errors and to ascending evidence of intact motor function, rather than direct transmission of motor-dysfunction predictions from the insula to SMA (11,18). Convergent functional magnetic resonance imaging, resting-state, and structural evidence supports SMA involvement (57).

The TPJ integrates motor intentions with sensory feedback to generate the sense of agency (58). Altered functional connectivity of the TPJ in FND (58) may disrupt the attribution of movements to volitional control, contributing to the experience of symptoms as involuntary through impaired integration of intended and observed motor output. Within the framework, this perceived involuntariness may then reinforce the dysfunction prior by being interpreted as evidence that the body is not under voluntary control.

Neuroimaging evidence converges across task-based, resting-state, and structural modalities (4,18,53,59,60) framed by Bègue *et al.* (61) as a “software and hardware problem” combining functional dysregulation and structural variation; per-study detail is presented in Supplementary 1d. Loss of sensory attenuation during self-generated movement (51) provides empirical support for altered sensorimotor prediction-error processing, and the conceptualization of FND as a dynamic lesion (62) synthesizes these findings consistently with the present account. Dissociative symptoms, which frequently co-occur with FND (63, 64, 65), may represent an extreme form of the agency disruption described above. Figure 1 summarizes the pathway from stress exposure to symptom generation. Alternative accounts, including constructed-emotion theory (66) and learning-based models, are compared with the present framework in Supplementary 3.

**Figure 1:**
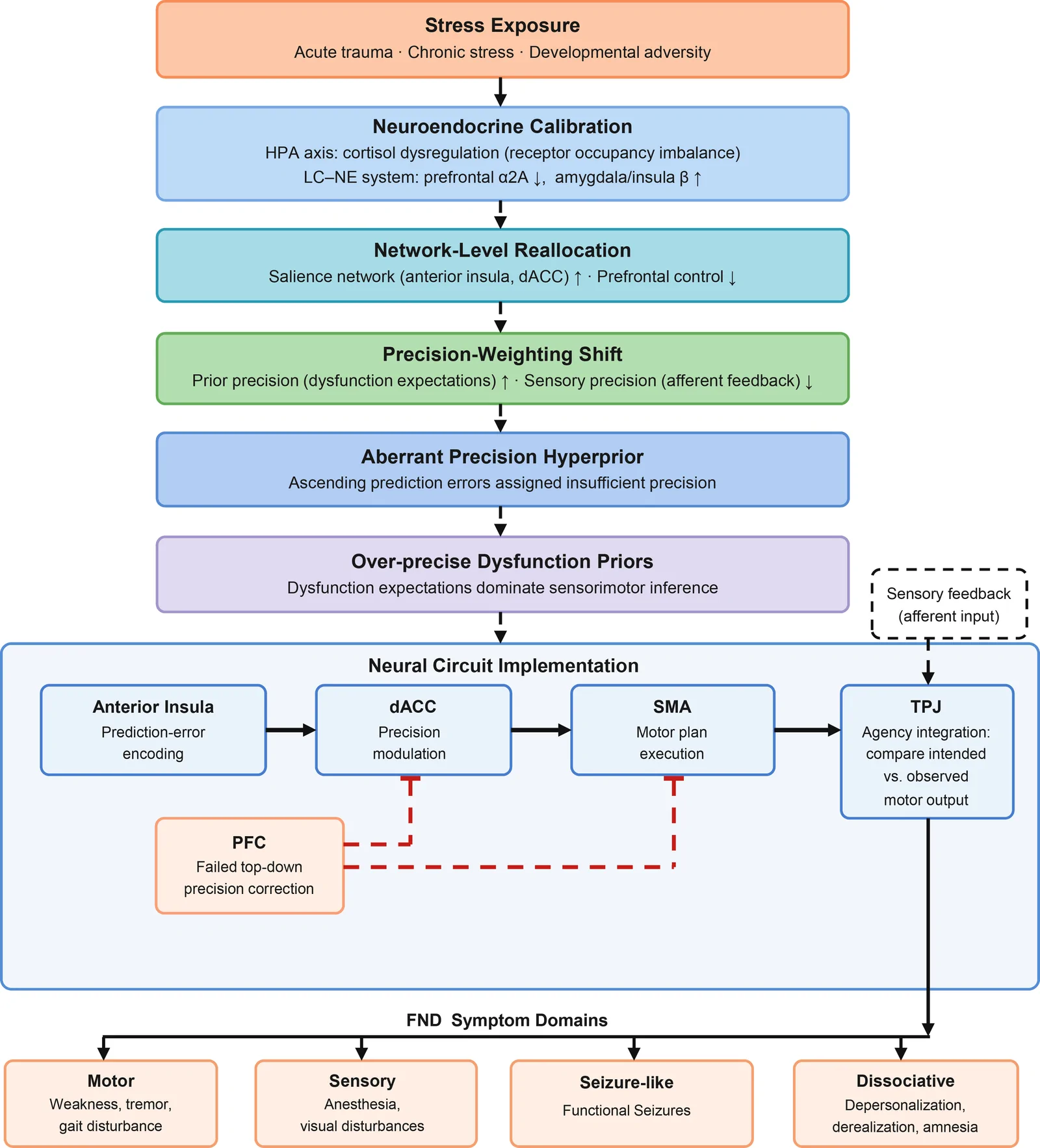
From stress exposure to sensorimotor dysfunction: a precision-weighting account of functional neurological disorder (FND). Stress exposure is associated with changes in neuroendocrine systems (hypothalamic-pituitary-adrenal axis [HPA], locus coeruleus–norepinephrine [LC–NE]), which are hypothesized here to alter precision weighting within salience and sensorimotor circuits rather than determining the content of dysfunctional predictions. This content is supplied by latent illness–related priors shaped by prior illness exposure and sociocultural illness representations; stress is proposed to increase the precision afforded to these priors while downweighting ascending corrective evidence. The figure depicts this proposed configuration, in which overprecise dysfunctional priors propagate through insula–dorsal anterior cingulate cortex (dACC)–supplementary motor area (SMA) circuits to produce FND symptoms across motor, sensory, seizure-like, and dissociative domains. The temporoparietal junction (TPJ) is shown as integrating intended motor output with afferent sensory feedback in generating the sense of agency. Solid black arrows denote established or empirically supported associations; black dashed arrows denote hypothesized mechanistic links within the proposed model that have not been directly demonstrated in FND; red dashed lines denote reduced or failed top-down precision correction. PFC, prefrontal cortex.

## Falsifiable Predictions

A persistent limitation of predictive processing accounts of psychopathology is that they often remain descriptively plausible without generating empirically disconfirmable predictions (67,68). The present account addresses this by deriving testable predictions at 5 levels of analysis, each with an explicit disconfirmation criterion. Table 2 summarizes these predictions, the methods required to test them, and the conditions that would count against the corresponding mechanistic claims.

## Clinical Implications

The framework proposes that interventions may alter symptoms by generating sensorimotor prediction errors capable of updating dysfunction priors. Reassurance and psychoeducation can provide an important conceptual foundation but, on this account, may not by themselves engage the sensorimotor processes hypothesized to maintain symptoms. Behavioral and physiotherapy-based interventions are therefore interpreted as potential routes to precision updating; whether their clinical effects are mediated by this mechanism remains to be established. Table 3 maps current and emerging treatment modalities to their proposed mechanisms, evidence base, and potential clinical implementation.

**Table 3:** Treatment Modalities for FND Mapped to Mechanism, Evidence Base, and Clinical Implementation

Treatment Modality	Mechanism Within Framework	Evidence Base	Clinical Implementation
Verbal Reassurance and Psychoeducation	Operates at the explicit-belief level only; does not generate sensorimotor prediction error and therefore does not reach the precision-weighted inference at which the dysfunction prior is encoded.	Low predicted efficacy as a standalone treatment; consistent with the clinical observation that reassurance alone does not resolve symptoms even when patients understand and accept the explanation.	Use as conceptual foundation paired with active prediction-error–generating interventions, not as a treatment in itself. Framing such as “the nervous system has learned a faulty prediction that can be retrained through specific movement experiences” provides a conceptual anchor that supports the active components below and reduces iatrogenic harm from competing biomedical explanations.
Cognitive Restructuring (CBT Verbal Component)	Targets explicit beliefs about symptoms but does not, on its own, generate the real-world prediction error required to update inference at the level at which the dysfunction prior operates.	Limited as a standalone target; typically incorporated into broader CBT packages that include behavioral experiments and graded exposure.	Limited as a standalone target. Deploy as one component of a CBT package alongside behavioral experiments and graded exposure to the symptom-related stimulus. Avoid relying on cognitive restructuring as the primary therapeutic mechanism.
CBT With Behavioral Experiments	Behavioral experiments generate real-world prediction errors that contradict the dysfunction prior; when these errors carry sufficient precision relative to the prior, they update the inference downstream.	Clinical evidence supports behavioral and physiotherapy-based approaches in FND, although whether their effects are mediated by prediction-error–driven precision updating has not been established (5,6).	Behavioral experiments should produce direct symptom violation (e.g., demonstrating preserved motor function under distraction or dual-task conditions) rather than abstract evidence gathering about beliefs. In-session experiments with immediate feedback are higher-fidelity than between-session assignments because the prediction error occurs in the presence of the clinician and can be processed in real time.
Physiotherapy With Distraction, Dual-Task Paradigms, Graded Motor Imagery, or Movement Retraining	Generates sensorimotor prediction error by producing movement under conditions in which the dysfunction prior does not generalize; the resulting mismatch between predicted and observed motor capacity updates the prior at the sensorimotor inferential level.	Clinical evidence supports specialist physiotherapy and movement-retraining approaches in motor FND (5,6,70); whether benefit depends specifically on prediction-error–driven precision updating has not been established.	Specialist FND physiotherapy following consensus protocols [Nielsen *et al.* recommendations (70)] is the first-line nonpharmacological treatment for motor FND. Key principles include diverting attention away from the affected limb during movement, using distraction or dual-task paradigms to facilitate movement outside habitual symptom-focused attention, demonstrating retained function to the patient, and progressively generalizing movement across contexts. Within the present framework, these strategies are interpreted as reducing the influence of dysfunction priors. Passive interventions such as massage and ultrasound, and sustained attention to the affected body part, are generally avoided because they may reinforce symptom-focused attention.
Pretreatment Precision Profiling (Interoceptive Decision Tasks and Resting-State Connectivity)	Estimates the prior-to-sensory precision ratio before treatment so that intervention intensity can be matched to the degree of entrenchment of overprecise priors, rather than determined by symptom severity alone.	Emerging; framework prediction yet to be tested in prospective stratification studies. Specified as the predictive (noncircular) row of Table 2.	Not yet ready for routine clinical use. In research-active centers, pretreatment fitting of a hierarchical Bayesian observer to a cardiac interoception task (Supplementary 4) alongside resting-state insula–dACC connectivity could be used to estimate baseline precision parameters; if validated, high prior precision and elevated salience-network connectivity may predict greater benefit from more intensive rehabilitation, whereas milder profiles may predict adequate response to outpatient physiotherapy.
Noninvasive Brain Stimulation (TMS or tDCS) Targeting Anterior Insula, dACC, or Dorsolateral Prefrontal Cortex	Inhibitory protocols (e.g., continuous theta burst stimulation) applied to salience nodes could reduce the precision assigned to dysfunction priors; excitatory protocols targeting dlPFC could enhance top-down correction of maladaptive predictions.	Hypothetical; existing neurostimulation studies in FND are small and heterogeneous and have not been guided by computational rationale for target selection (71). Theta burst TMS over the right TPJ has produced neural changes in a region critical to agency processing (72), providing initial proof of concept.	Not ready for routine clinical use. Reserve for research protocols with explicit computational rationale for target selection and explicit preregistration of effect size criteria. Future research should test the framework-derived hypotheses against control conditions and should measure precision parameters before and after stimulation to confirm that any clinical effect is mediated by the proposed mechanism rather than by nonspecific arousal effects.

The table distinguishes treatments by their proposed mechanism within the present framework, by the maturity of their evidence base, and by principles of clinical implementation. Established indicates interventions with existing clinical evidence consistent with the framework. Emerging indicates interventions where the framework generates a specific testable prediction not yet tested in dedicated studies. Hypothetical indicates speculative directions for future intervention research that should not yet enter routine clinical use.

CBT, cognitive behavioral therapy; dACC, dorsal anterior cingulate cortex; dlPFC, dorsolateral prefrontal cortex; FND, functional neurological disorder; tDCS, transcranial direct current stimulation; TMS, transcranial magnetic stimulation; TPJ, temporoparietal junction.

The framework also makes a specific discriminative prediction. If confirmed, attenuated insula prediction-error signaling during voluntary movement would provide a candidate neural marker for differentiating FND from somatization and health anxiety at the circuit level. This prediction sits alongside the predictions in Table 2 as a falsifiable empirical commitment and, if replicated prospectively, could inform future mechanism-based treatment selection.

## Limitations and Future Directions

The framework’s central inferential step has not been established. No study has directly shown that stress-related neuroendocrine or neuromodulatory changes produce measurable changes in precision weighting within sensorimotor hierarchies. Blood oxygen level–dependent (BOLD) signal, cortisol indices, and receptor pharmacology should likewise not be treated as direct measures of Bayesian precision. The proposed mapping between these biological processes and precision weighting is therefore a mechanistic hypothesis that organizes existing findings and generates disconfirmable predictions, rather than a description of a demonstrated pathway. Further methodological constraints on this inferential structure, including reverse inference and circularity, are detailed in Supplementary 5. Current evidence is cross-sectional; longitudinal designs assessing computational parameters before and after stress exposure (e.g., military personnel before and after deployment, surgical cohorts at risk of FND) would be required to test whether stress causally produces the proposed precision changes. The neuroimaging evidence supporting the proposed circuit architecture derives predominantly from motor FND and functional seizures; whether the same dynamics operate in functional sensory symptoms or persistent perceptual-postural dizziness remains to be established across subtypes. The present framework does not specify how these mechanisms apply to the pain and fatigue commonly comorbid with FND. Because allostatic perturbation can be realized through degenerate brain-body configurations (10), the predicted chronic hypocortisolism profile may characterize only a subset of FND presentations.

The comorbidity challenge is substantial and currently constrains what the framework can claim. Rates of comorbid depression, anxiety, and PTSD in FND range from 30% to 80% (8), making samples without psychiatric comorbidity difficult to recruit, and almost all cited supporting evidence is drawn from samples in which such comorbidities are common. Whether precision miscalibration is specific to FND, rather than a transdiagnostic feature also present in chronically stressed psychiatric samples, is therefore currently unestablished; the framework’s specificity claim, that the shared precision pathology is localized to specific sensorimotor and interoceptive channels in FND, is an empirical claim to be tested rather than a property the existing evidence supports. Testing it requires 3 groups: patients with FND, psychiatric control participants with comparable stress exposure but no functional symptoms, and healthy control participants. If the precision parameters described in Table 2 differentiate FND from psychiatric control participants, this would support specificity of the predicted precision pattern to FND; if not, the account would require transdiagnostic revision.

## Conclusions

Predictive-processing accounts of FND and research on stress neurobiology have largely developed in parallel. The present synthesis links these literatures. It specifies a candidate pathway by which allostatic perturbation may miscalibrate precision and proposes that the resulting imbalance is expressed in sensorimotor and interoceptive channels rather than through pathology unique to FND. This integration is more than redescription because it yields a prediction neither parent literature makes alone: attenuation of insula prediction-error signaling during movement that scales with stress-system dysregulation and may ease under acute reduction of arousal, a discriminative prediction whose support depends on convergence between the neural, computational, and arousal-dependent patterns specified in Table 2 and whose failure would weaken the account. The same logic reframes treatment, offering a possible account of why reassurance alone may be insufficient and why interventions generating sensorimotor prediction error may succeed, while pointing toward treatment selection by computational profile rather than diagnostic label. The account therefore provides a mechanistic, testable link between psychological precipitants and neural expression in FND.
