# Supplementary material for: Functional Neurological Disorder as a Disorder of Sensorimotor Inference Under Stress: A Neurocomputational Account
Source: Biol Psychiatry Glob Open Sci. 2026 Sep 9;7(1):100825. doi: 10.1016/j.bpsgos.2026.100825 (PMC13691508; doi:10.1016/j.bpsgos.2026.100825)
Supplement: Supplementary file 1 [file mmc1.pdf]

SUPPLEMENTARY INFORMATION

Functional Neurological Disorder as a Disorder of Sensorimotor Inference Under Stress: A Neurocomputational Account

Putica

Supplementary Materials

Supplementary materials for "Functional Neurological Disorder as a Disorder of Sensorimotor Inference Under Stress: A Neurocomputational Account." The sections below provide formal, methodological, and comparative detail designed to complement the main review while preserving a concise main-text narrative.

Supplementary 1

Detailed Mechanism Elaborations

The following subsections provide elaborated detail for the receptor-level, network-level, neural-implementation, and clinical-implication content of the main text. The main text presents compressed versions of these claims; the full elaborations, with the specific mechanistic chains and clinical hypotheses they generate, are presented here.

1a. Receptor-Level Mechanisms

The convergence between neuroendocrine and receptor-level changes and the computational consequence of prior-dominant inference is a mechanistic hypothesis rather than a demonstrated empirical link. The receptor findings establish that the relevant stress pharmacology is altered, and the clinical data establish that HPA dysregulation is present in FND, but neither establishes on its own that these changes produce prior-dominant inference, and HPA dysregulation is itself transdiagnostic rather than specific to FND. On the present account, specificity arises not from the endocrine disturbance but from the sensorimotor locus at which precision is recalibrated, and the proposed cortisol-to-computation bridge is rendered testable by the prediction that neuroendocrine indices should correlate with computational precision parameters within FND (Table 2; in manuscript).

Cortisol acts through high-affinity mineralocorticoid receptors (MR) and low-affinity glucocorticoid receptors (GR), which are co-expressed in limbic regions and mediate complementary, time- and dose-dependent actions rather than separate or opposing ones (1). Predominant MR occupancy under basal and early-stress conditions supports appraisal, response selection, and flexible encoding, whereas recruitment of GR as cortisol rises promotes

consolidation and behavioural adaptation. Chronic stress is thought to disturb this MR/GR balance, and it is the disruption of their coordinated action, rather than the isolated activity of either receptor, that is associated with impaired behavioural adaptation and heightened vulnerability to stress-related conditions (1). In computational terms, the present account proposes that such a shift in MR/GR balance corresponds to reduced precision on corrective evidence and greater weight on entrenched priors; consistent with the framing above, this mapping is an interpretation rather than a relationship demonstrated in the cited endocrinological literature.

Norepinephrine provides a parallel route. Prefrontal α2A-adrenergic receptors support stable top-down prediction, whereas arousal-driven noradrenergic signalling in amygdala and insula increases the gain on salient, high-priority signals while suppressing competing low-priority inputs, rather than amplifying ascending evidence uniformly. Chronic stress is proposed to downregulate prefrontal α2A control and to sensitise this arousal-driven selectivity. These mechanisms alter gain rather than content; which representations are prioritised is attributed here to latent illness-related priors and bodily attention. In the present account, reduced weighting of corrective proprioceptive or exteroceptive evidence is therefore not treated as a direct consequence of noradrenergic receptor physiology, but as a hypothesised downstream result when dysfunction-related priors and body-focused attention already bias which representations enter high-priority competition.

Two further points elaborate the main-text treatment of HPA dynamics. First, on the persistence of stress-related HPA change: early-life adversity has been associated with lasting alterations in diurnal cortisol activity in adulthood, indicating that stress-related HPA changes can persist well beyond the period of exposure. It is therefore possible, though not established,

that such persistence contributes to the well-documented chronicity of FND symptoms, in which symptoms frequently outlast the precipitating stressor. Second, on the limits of current evidence: the available cortisol data in FND are cross-sectional and do not track cortisol across the resolution of a stressor, so they cannot establish whether the neuroendocrine changes precede, accompany, or follow symptom onset. Testing the proposed causal sequence would require longitudinal designs that assess diurnal cortisol profiles and computational precision parameters at multiple timepoints in at-risk cohorts (for example, surgical or post-injury cohorts, or military personnel assessed before and after deployment), so that the temporal relationship between HPA change and the emergence of over-precise dysfunction priors can be observed directly rather than inferred.

1b. Network-Level Reallocation: Extended Detail

The configuration described in the main text, salience-network activity amplified and prefrontal engagement reduced, is proposed to shift the arbitration between high-precision priors and corrective ascending evidence such that priors carry greater weight in inference while the contribution of corrective evidence is diminished. In FND, when bodily sensations are ambiguous, this stress-biased configuration is proposed to favour dysfunction-congruent interpretations, with reduced prefrontal updating sustaining them. Neuroimaging findings in FND corroborate this pattern, showing elevated salience-network activation and reduced default-mode and executive-network engagement during symptom provocation (2). Converging evidence comes from PTSD, in which the same neuroendocrine pathways produce stress-calibrated priors about bodily threat rather than bodily dysfunction (3–6); the shared mechanism is precision miscalibration, while the distinguishing feature is the sensorimotor locus of the over-precise priors in FND.

1c. The Interoceptive Channel Contribution

A complementary interoceptive contribution operates within the same precision framework rather than as a separate route. Visceral arousal arising from affective states (cardiac, respiratory, and thermal changes) is conveyed by interoceptive afferents, and repeated mislabelling of this arousal as somatic dysfunction is here understood as the learning, with disproportionate precision, of interoceptive priors predicting bodily pathology (7). On the present account this is the interoceptive expression of the same precision pathology, governed by the same hyperprior structure that governs the proprioceptive and motor channels, and is most relevant to symptom presentations with prominent visceral or autonomic features. Constructed-emotion accounts, which place a similar emphasis on the learned interpretation of arousal, propose a partly different generative mechanism and are addressed alongside other alternative models in Supplementary 3 (8).

1d. Neuroimaging Detail

The main text gives a high-level summary of the neuroimaging evidence supporting the proposed circuit architecture. The per-study detail is presented here. Aybek et al. (9) demonstrated that FND patients exhibit limbic hyperactivation, particularly in the amygdala and insula, during emotional processing, with aberrant functional connectivity between limbic and motor regions even in response to positively valenced stimuli. Hassa et al. (10) reported increased amygdala activation during cognitive reappraisal in FND, consistent with persistent limbic engagement during attempted regulation. Together, these findings are consistent with disrupted insula–limbic integration contributing to altered emotion–motor coupling.

The dACC has been implicated in modulating precision under uncertainty (11, 12), and altered dACC engagement during affective processing (13) and ACC hypometabolism at rest in functional seizures (14) are consistent with altered engagement of regions implicated in precision-related processing. Reduced coupling between prefrontal control regions and sensorimotor cortices (15, 16) is consistent with reduced top-down correction of dysfunction priors operating downstream.

In FND, high-precision dysfunction priors arriving at the SMA override proprioceptive feedback that would normally indicate intact motor capacity; voluntary movement is suppressed in a manner consistent with the SMA implementing a dysfunction-congruent prediction. Convergent evidence across task-based fMRI, resting-state, and structural imaging modalities supports SMA involvement (17, 18). At the structural level, altered white matter microstructure has been reported in tracts connecting insula, cingulate, and motor regions, and Bègue et al. characterised the combination of functional dysregulation and structural variation as a "software and hardware problem." Loss of sensory attenuation during self-generated movement provides empirical support for altered sensorimotor prediction-error processing, and the conceptualisation of FND as a "dynamic lesion" synthesises these task-based, resting-state, and structural findings within a framework consistent with the present account.

Interoceptive fMRI paradigms reveal altered insula activation during visceral prediction tasks (19). Neuroendocrine data complement these findings: a flattened cortisol awakening response has been observed in FND, and cortisol amplitude is associated with clinical outcome (20), providing preliminary support for the physiological prediction in Table 2.

Supplementary 2

Locus Determination: Extended Evidence for Three Candidate Factors

The main text identifies three candidate factors that may determine why stress-driven precision miscalibration localises to sensorimotor circuits in FND rather than to affective or threat-detection systems. Extended evidence and testable predictions for each factor are provided below.

Factor 1: Prior Sensorimotor Experience as a Template

FND onset frequently follows minor physical events such as a stumble, a mild injury, a surgical procedure, or a period of immobility (21). On the present account, the event itself does not implant a dysfunction prior in sensorimotor circuits. Prior content is supplied by pre-existing illness representations built up through previous illness experience in the self or in observed others, including sociocultural illness expectations, while the physical event acts as a salience-amplifying trigger that draws pathological attention to the body and increases the precision afforded to those latent priors within the relevant sensorimotor channel. Hallett et al. (21) noted that FND commonly co-occurs with or is triggered by such pathophysiological events. Where the event also generates a transient sensorimotor prediction of dysfunction, that prediction would normally be revised as the body recovers; if stress-driven neuromodulation inflates the precision afforded to the latent prior during or shortly after the event, however, the salience-driven precision uplift can consolidate the latent prior into an over-precise prior.

The sensorimotor locus is thus not arbitrary but is shaped by which circuits drew pathological attention during the precipitating experience. This account is consistent with the clinical observation that functional weakness often affects a limb that was previously injured and that functional seizures frequently emerge in individuals with a history of epilepsy or head injury

(21), because the prior physical experience establishes the latent template whose precision stress subsequently amplifies.

Testable prediction: In prospective samples (e.g., post-surgical cohorts), patients who develop FND should show precision parameter changes localised to the sensorimotor circuits corresponding to the body region affected by the precipitating physical event. Patients who experience the same physical event but develop anxiety or PTSD instead should show precision changes in threat-detection circuits without sensorimotor specificity.

Factor 2: Structural Connectivity Predisposition

Individual variation in the functional integration of limbic, salience, and motor circuits is likely to influence whether stress-related precision recalibration is expressed predominantly in affective or sensorimotor channels. Existing neuroimaging evidence in FND includes diffusion tensor imaging of white matter microstructure in tracts connecting insula, cingulate, and motor regions (22) and a meta-analysis of motor conversion disorder reporting alterations in prefrontal and motor circuits (23), alongside broader pathophysiological accounts that posit bridging variables between symptoms and circuits (24); these reports concern functional and, in places, structural measures whose relationship to symptom onset is not yet established. Whether such variation, including potential structural contributions, predisposes specifically to sensorimotor rather than affective entrenchment is a prediction of the present framework rather than an established finding, and is identified as a target for future investigation.

Drane et al. (24) proposed a pathophysiology-focused framework identifying five interacting constructs in FND (emotion processing, agency, attention, interoception, and predictive inference) and called for research characterising the neuroendocrine, autonomic, and structural variables that bridge symptoms and circuits. Individual variation in insula–SMA

pathway connectivity is one such variable. People with stronger insula–motor coupling may be more vulnerable to sensorimotor hyperprior entrenchment under stress, while those with stronger amygdala–prefrontal coupling may instead develop affective hyperpriors that manifest as anxiety or PTSD.

Testable prediction: In at-risk populations (e.g., individuals with high adverse childhood experience scores), baseline DTI measures of insula–SMA tract integrity should predict subsequent development of FND (rather than PTSD or depression) following a stressful life event. This requires prospective structural imaging in longitudinal designs.

Factor 3: Interoceptive-Neuroendocrine Vulnerability Profile

Individual differences in interoceptive processing style and neuroendocrine regulation may shape susceptibility to stress-related precision recalibration. Weber et al. (25) identified biopsychological trait markers in a large FND sample (N = 86 patients, 76 controls), observing a flattened cortisol awakening response alongside hippocampal volume reductions and preceding childhood trauma. These alterations were characterized as trait markers that predated the current episode. The present account treats such neuroendocrine variation as one plausible vulnerability factor that may shape susceptibility to stress-related precision recalibration; the cited evidence does not, however, establish within-individual cortisol-to-brain coupling or specificity to sensorimotor integration circuits, and these remain open questions to be tested.

On the present account, individuals whose neuroendocrine profiles preferentially affect sensorimotor integration circuits, rather than amygdala-centred threat circuits, would be expected to develop over-precise dysfunction priors in motor and proprioceptive domains; this is a prediction of the framework rather than an established pattern. Together, the three factors are

proposed to interact in determining why particular individuals develop FND rather than another stress-linked condition.

Testable prediction: Within FND samples, cortisol awakening response profiles should correlate more strongly with sensorimotor precision parameters (from Table 2's algorithmic-level task) than with affective precision parameters. In psychiatric control groups (PTSD, depression), the reverse pattern should hold: cortisol profiles should correlate with affective/threat-related precision parameters but not sensorimotor ones.

Supplementary 3

Extended Neural Mapping and Competing Models

The main manuscript focuses on an anterior insula–dACC–SMA circuit because it is the most direct pathway from precision-weighted inference to motor output. That simplification is deliberate, and other regions are also likely to be involved. This section addresses the broader neural context and contrasts the proposed account with competing models.

Within the present framework, the anterior insula encodes interoceptive prediction errors and constructs a current body-state representation, the dorsal anterior cingulate modulates the precision assigned to those signals under uncertainty, and the supplementary motor area implements the resulting inference in the motor domain. Stress-induced dysregulation of this circuit biases the system toward dysfunction expectations.

The thalamus is relevant because it gates ascending visceral and somatosensory input before cortical integration. The posterior insula is relevant because it represents more primary interoceptive signals before these are integrated into higher-order body-state inference in anterior insula. The cerebellum may also be relevant insofar as it contributes to forward modeling, prediction, and sensorimotor error correction. These regions do not change the paper's main claim, but they identify directions for future work and acknowledge that the core mapping is simplified.

The model can also be tested against alternatives. A sensory-gain account would predict that abnormal symptoms arise because sensory input is amplified rather than discounted. Under that model, one would expect heightened rather than attenuated prediction-error signaling in anterior insula during tasks that generate proprioceptive mismatch. An attentional-capture account would predict that the core problem is abnormal allocation of attention rather than

precision weighting, producing relatively normal sensory prediction-error signaling but altered prefrontal gating. By contrast, the hyperprior account predicts that dysfunction expectations dominate inference and therefore that error signals inconsistent with those expectations will be attenuated.

These alternative patterns are empirically distinguishable. Event-related functional neuroimaging during voluntary movement with proprioceptive perturbation should reveal one of three signatures: reduced prediction-error signaling if the hyperprior account is correct, increased signaling if a sensory-gain account is correct, or relatively preserved signaling with altered control-network modulation if an attentional account is correct. What distinguishes this model is that it makes a specific prediction that competing models do not share.

Detailed Comparison with Alternative Accounts

The following compares the present account with alternative models of FND.

Predictive processing framework (7). Edwards et al. established the foundational predictive processing account, showing that hyperprecise priors can override sensory evidence to produce functional symptoms. Their account does not, however, specify why priors become hyperprecise, predict which stress-exposed individuals develop FND, or generate measurable biomarkers for treatment stratification. The present account retains that computational architecture but specifies the biological mechanism upstream. The distinguishing prediction is that neuroendocrine indices (cortisol diurnal slope, HRV) should correlate with precision parameters; if they do not, the stress-calibration mechanism lacks support even if the downstream precision account remains valid.

Constructed emotion theory (8). Jungilligens et al. applied constructed emotion theory to FND, emphasising allostatic disruption and emotion category construction. Their account

operates primarily at the level of affective inference rather than sensorimotor prediction, and does not explain why the outcome is motor dysfunction rather than persistent emotional distress. The distinguishing test is the discriminative prediction: attenuated insula prediction-error signalling during voluntary movement (hyperprior account) versus altered affective-interoceptive processing without motor-specific disruption (constructed emotion account).

Learning-based accounts. Conditioning and reinforcement models propose that FND symptoms are acquired through associative learning or maintained through avoidance. These accounts explain some features of FND but do not explain why precision imbalances localise to motor systems, why symptoms persist without reinforcement, or why distraction produces symptom variability. The present account incorporates physical events as templates for dysfunction priors while providing the computational mechanism that learning-based models lack.

Supplementary 4

Task Design and Model-Fitting Strategy

This section describes how that could be done in practice. The goal is not to prescribe a single task, but to show that the model's parameters can be estimated independently rather than inferred from symptoms alone. One suitable paradigm is a cardiac interoception task in which participants judge whether externally delivered tactile pulses are synchronous with their heartbeat. Sensory precision is manipulated by varying the temporal jitter between the pulse and the cardiac signal. Prior precision is manipulated by presenting contextual cues that probabilistically signal whether synchrony is likely to be high or low on a given trial. Participants provide a binary synchrony judgment and a confidence rating on each trial. As the task varies sensory evidence and expectation independently, it can estimate how strongly each influences perception

Responses can be fitted using hierarchical Bayesian observer models. At the participant level, the model estimates how much weight is placed on prior expectations, how much reliability is assigned to sensory evidence, and how quickly beliefs are updated across trials. Model comparison can then be performed using standard Bayesian procedures, such as variational Bayes or leave-one-out predictive fit, to determine whether models with flexible precision parameters outperform simpler alternatives that assume fixed weighting.

The central prediction is straightforward, and is presented here as a replication target rather than as a discriminative test of the stress-calibrated account. Relative to controls, individuals with FND should show elevated prior precision and reduced sensory precision, particularly when task conditions require belief updating in the face of contradictory bodily evidence. These parameters should correlate with symptom severity and with circuit-level

indices such as insula–supplementary motor area or insula–dorsal anterior cingulate connectivity. These FND-versus-control contrasts are entailed by the general Bayesian and precision-imbalance accounts of FND already established in the literature; they are therefore relocated here from the main-text prediction table, where only within-FND, arousal-dependent, and prospective relationships specifically entailed by the stress-calibration mechanism are retained (see main text, Table 2). This approach separates distinct explanations for impaired performance. Poor interoceptive accuracy could arise because sensory channels are noisy, because priors are over-precise, or because both are true. The model is useful precisely because it distinguishes among these possibilities.

The same logic can be extended beyond cardiac tasks. Proprioceptive perturbation paradigms, intentional binding tasks, and paradigms that experimentally manipulate expectations about bodily capability could all be fitted within the same inferential framework. What matters is that the task separates expectation from sensory evidence, and that trial-by-trial responses allow their relative influence to be estimated.

The key point is that the proposed theory is not merely post hoc. It generates a concrete empirical program in which the central computational claim, that over-precision of dysfunction priors relative to sensory evidence, can be tested quantitatively. This is especially important because one of the major weaknesses of many predictive processing accounts is that they remain verbally plausible without demonstrating how their putative parameters would be estimated in practice. The present framework avoids that problem by specifying how the parameters would actually be estimated.

Replication Targets Relocated from the Main-Text Prediction Table

Two predictions that appeared in earlier versions of the main-text prediction table are presented here as replication targets in the same cohort, rather than as discriminative tests of the present account. Both are consistent with the general precision-imbalance account of FND already established in the literature and would be predicted by any Bayesian account of functional symptoms, with or without a stress component; they are therefore not diagnostic of the stress-calibration mechanism specifically, and are retained here as confirmatory benchmarks that the cohort and task perform as expected before the stress-specific within-FND, arousal-dependent, and prospective relationships in Table 2 are evaluated.

The first is an algorithmic-level replication target: relative to matched healthy controls, individuals with FND should show a higher prior-to-sensory precision ratio (elevated prior precision and reduced sensory precision) on the cardiac interoception task described above, with the difference most pronounced under conditions requiring belief updating against contradictory bodily evidence. The second is a neural-implementation replication target: relative to matched healthy controls, individuals with FND should show altered insula–dorsal anterior cingulate resting-state connectivity. A failure to replicate either FND-versus-control contrast would call into question the general precision-imbalance account of FND on which the present framework builds, but would not, on its own, disconfirm the stress-calibration mechanism, whose specific predictions are the within-FND covariations, arousal-dependent modulation, and prospective treatment-response relationship set out in Table 2.

Supplementary 5

Operational Mapping and Limits of Inference

The framework's discriminative value depends on the mapping between computational constructs (precision, priors, prediction errors) and measurable signals, and that mapping is not direct. Precision is not directly observable, and BOLD responses index a confound-laden mixture of metabolic demand, attention, arousal, and baseline activity, so the inference from a regional BOLD signal to a specific computational parameter is indirect and admits alternative explanations. Three constraints address this. First, no single measurement modality is treated as decisive: the discriminative claim requires convergence across an algorithmic-model parameter (sensory-precision parameter from a hierarchical Bayesian observer fit), a neural signature (attenuated anterior insula response during proprioceptive perturbation), and an arousal-dependent modulation (partial restoration under β-adrenergic blockade). Failure of an individual component would weaken the corresponding mechanistic claim, whereas failure of the predicted cross-modal convergence would count more strongly against the integrated account. Second, precision is constrained to specific channels and intermediate hierarchical levels rather than invoked as a general-purpose variable: content is attributed to latent illness-related priors, and neuroendocrine systems are assigned a role in modulating precision rather than in specifying prediction content. Third, the BOLD-based predictions are interpreted within the known limits of fMRI inference: an attenuated insula response is compatible with reduced ascending precision but also with attention, arousal, or neurovascular confounds, and the convergence requirement in the discriminative row of Table 2 is what licenses the precision interpretation against these alternatives.

Two related interpretive risks follow from these limits. Neuroimaging predictions face known reverse-inference limitations: a finding that a region shows the predicted activation does not, on its own, license the inverse claim that the computational construct is implemented in that region (26, 27). The convergence requirement across modalities, rather than the BOLD finding alone, is what carries the inferential weight. The second risk is circularity: if the hyperprior is inferred from the symptoms it is then used to explain, the account becomes unfalsifiable. The convergence-based falsification logic guards against this by requiring predictions that go beyond post hoc consistency with the symptoms. A specifically non-circular prediction is also included in Table 2 (the “Predictive” row), in which baseline computational precision parameters are required to predict subsequent treatment response after adjustment for baseline symptom severity; this requires the precision measurement to capture variance that is not itself downstream of current symptoms. Behavioural paradigms index downstream inference consequences without isolating latent parameters, and the experimental paradigms specified in Table 2 are designed to manipulate precision experimentally rather than to infer it post hoc; the computational modelling approach extracts latent parameters that are not directly observable in behaviour alone.

The same constraints apply to the stress-to-precision claim itself. The cited literature directly supports stress effects on arousal, salience processing, prefrontal function, and the neuromodulators that act on synaptic gain. The inferential step from these effects to a shift in hierarchical Bayesian precision is theoretical rather than evidential: it depends on the standard predictive-coding interpretation in which neuromodulator-driven changes in synaptic gain implement precision, which is well developed in the computational literature but has not been directly demonstrated for stress-driven changes specifically. The present account is therefore best

understood as a theoretical proposal articulating how known stress effects could produce changes in hierarchical Bayesian precision, rather than as empirical evidence that they do so. The intermediate chain runs from stress, through neuromodulator changes (cortisol acting via mineralocorticoid and glucocorticoid receptors; noradrenaline acting via α2A and β-adrenergic receptors), through changes in synaptic gain in the relevant pyramidal cell populations, to the predictive-coding interpretation of those gain changes as precision, to the testable consequences specified in Table 2. The first three steps are empirically established, the fourth is a theoretical mapping in predictive coding, and the fifth is what the Table 2 predictions are designed to test. Conversion of the analogical bridge into an evidential one therefore depends on the within-FND covariation between stress variables and computational precision parameters specified in the algorithmic, neural-implementation, and physiological rows of Table 2; absence of those covariations would indicate that the analogical inference does not hold for stress-driven changes specifically, even if the underlying neuropharmacology is preserved. Quantitative thresholds for confirmation and disconfirmation of the Table 2 predictions are not specified in advance, because no prior data exists to anchor effect-size expectations for these novel predictions. Empirical work testing the framework should pre-register effect-size criteria and statistical thresholds, so that the convergence requirement specified above is evaluated against predetermined rather than post hoc standards.

A related question concerns whether the framework is generative in the sense of producing predictions that existing evidence could not already accommodate. A distinction is needed here. The FND-versus-control contrasts (elevated prior precision and reduced sensory precision relative to controls, and altered insula–dACC connectivity relative to controls) are entailed by the general precision-imbalance account of FND and are not discriminating; they are

presented as replication targets in Supplementary 4. The algorithmic, neural-implementation, and physiological predictions in Table 2, by contrast, test within-FND covariation between stress variables and computational, connectivity, or physiological measures, and these covariations are not predicted by precision-coding or stress-neurobiology accounts that contain no stress term, so they are generative relative to those parent accounts rather than post hoc accommodations of them. The discriminative prediction is generative in a further sense, in that it separates the present account from competing precision-coding models. The joint pattern it specifies (stress-stratified insula prediction-error attenuation acutely modulated by β-adrenergic blockade and converging with the algorithmic-row precision parameter pattern) is not predicted by pure sensory-gain, attentional-capture, or stress-independent Bayesian accounts. Its viability depends on three explicit assumptions: that insula BOLD during proprioceptive perturbation indexes prediction-error processing; that the effects of β-blockade on the predicted neural signature can be distinguished from accompanying changes in heart rate, peripheral autonomic feedback, subjective arousal, attentional state, and symptom-related anxiety; and that BOLD attenuation can be cross-validated by the algorithmic-row model parameter pattern. Each is independently testable.

A further constraint specific to precision-coding accounts is that prior precision, likelihood precision, and attentional precision are not cleanly separable in BOLD (28): attenuated insula prediction-error signaling can arise either from reduced sensory precision on the perturbation channel or from elevated attentional precision on a competing channel. The convergence requirement in Table 2 is what specifies which of these the framework's prediction commits to, by pairing the BOLD signature with an algorithmic-row precision parameter pattern

and a behavioural signature that the elevated-competing-channel-attention account does not predict.

References

1. de Kloet ER (2014): From receptor balance to rational glucocorticoid therapy. Endocrinology 155: 2754–2769. https://doi.org/10.1210/en.2014-1048
2. Perez DL, Nicholson TR, Asadi-Pooya AA, Bègue I, Butler M, Carson AJ, et al. (2021): Neuroimaging in functional neurological disorder: State of the field and research agenda. NeuroImage Clin 30: 102623. https://doi.org/10.1016/j.nicl.2021.102623
3. Cooper SE, van Dis EAM, Hagenaars MA, Krypotos AM, Nemeroff CB, Lissek S, et al. (2022): A meta-analysis of conditioned fear generalization in anxiety-related disorders. Neuropsychopharmacology 47: 1652–1661. https://doi.org/10.1038/s41386-022-01332-2
4. Putica A, Argus A, Khanna R, Nursey J, Varker T (2025): Interoceptive interventions for posttraumatic stress: A systematic review of treatment and interoception outcomes. Traumatology 31: 195–211. https://doi.org/10.1038/s44159-025-00479-1
5. Kube T, Berg M, Kleim B, Herzog P (2020): Rethinking post-traumatic stress disorder: A predictive processing perspective. Neurosci Biobehav Rev 113: 448–460. https://doi.org/10.1016/j.neubiorev.2020.04.014
6. Putica A, Felmingham KL, Garrido MI, O'Donnell ML, Van Dam NT (2022): A predictive coding account of value-based learning in PTSD: Implications for precision treatments. Neurosci Biobehav Rev 138: 104704. https://doi.org/10.1016/j.neubiorev.2022.104704
7. Edwards MJ, Adams RA, Brown H, Parées I, Friston KJ (2012): A Bayesian account of 'hysteria.' Brain 135: 3495–3512. https://doi.org/10.1093/brain/aws129
8. Jungilligens J, Paredes-Echeverri S, Popkirov S, Barrett LF, Perez DL (2022): A new science of emotion: Implications for functional neurological disorder. Brain 145: 2648–2663. https://doi.org/10.1093/brain/awac204
9. Aybek S, Nicholson TR, O'Daly O, Zelaya F, Kanaan RA, David AS (2015): Emotion–motion interactions in conversion disorder: An fMRI study. PLoS ONE 10: e0123273. https://doi.org/10.1371/journal.pone.0123273

10. Hassa T, Spiteri S, Schmidt R, Merkel C, Schoenfeld MA (2021): Increased amygdala activity associated with cognitive reappraisal strategy in functional neurologic disorder. Front Psychiatry 12: 613156. https://doi.org/10.3389/fpsyt.2021.613156

11. Cole N, Harvey M, Myers-Joseph D, Gilra A, Khan AG (2024): Prediction-error signals in anterior cingulate cortex drive task-switching. Nat Commun 15: 7088. https://doi.org/10.1038/s41467-024-51368-9

12. Vriens T, Vassena E, Pezzulo G, Baldassarre G, Silvetti M (2025): Meta-Reinforcement Learning reconciles surprise, value, and control in the anterior cingulate cortex. PLoS Comput Biol 21: e1013025. https://doi.org/10.1371/journal.pcbi.1013025

13. Pick S, Goldstein LH, Perez DL, Nicholson TR (2019): Emotional processing in functional neurological disorder: A review, biopsychosocial model and research agenda. J Neurol Neurosurg Psychiatry 90: 704–711. https://doi.org/10.1136/jnnp-2018-319201

14. Arthuis M, Micoulaud-Franchi JA, Bartolomei F, McGonigal A, Guedj E (2015): Resting cortical PET metabolic changes in psychogenic non-epileptic seizures (PNES). J Neurol Neurosurg Psychiatry 86: 1106–1112. https://doi.org/10.1136/jnnp-2014-309390

15. Voon V, Brezing C, Gallea C, Ameli R, Roelofs K, LaFrance WC, et al. (2010): Emotional stimuli and motor conversion disorder. Brain 133: 1526–1536. https://doi.org/10.1093/brain/awq054

16. van der Kruijs SJM, Bodde NMG, Vaessen MJ, Lazeron RHC, Vonck K, Boon P, et al. (2012): Functional connectivity of dissociation in patients with psychogenic non-epileptic seizures. J Neurol Neurosurg Psychiatry 83: 239–247. https://doi.org/10.1136/jnnp-2011-300776

17. Baizabal-Carvallo JF, Hallett M, Jankovic J (2019): Pathogenesis and pathophysiology of functional (psychogenic) movement disorders. Neurobiol Dis 127: 32–44. https://doi.org/10.1016/j.nbd.2019.02.013

18. Begue I, Adams C, Stone J, Perez DL (2019): Structural alterations in functional neurological disorder and related conditions: A software and hardware problem? NeuroImage Clin 22: 101798. https://doi.org/10.1016/j.nicl.2019.101798

19. Sojka P, Serranová T, Khalsa SS, Perez DL, Diez I (2024): Altered neural processing of interoception in patients with functional neurological disorder: A task-based fMRI study. J Neuropsychiatry Clin Neurosci 37: 149–159. https://doi.org/10.1176/appi.neuropsych.20240070

20. Weber S, Bühler J, Messmer F, Bruckmaier R, Aybek S (2024): Cortisol in functional neurological disorders: State, trait and prognostic biomarkers. J Psychosom Res 179: 111615. https://doi.org/10.1016/j.jpsychores.2024.111615

21. Hallett M, Aybek S, Dworetzky BA, McWhirter L, Staab JP, Stone J (2022): Functional neurological disorder: New subtypes and shared mechanisms. Lancet Neurol 21: 537–550. https://doi.org/10.1016/S1474-4422(21)00422-1

22. Sojka P, Diez I, Bareš M, Perez DL (2021): Individual differences in interoceptive accuracy and prediction error in motor functional neurological disorders: A DTI study. Hum Brain Mapp 42: 1434–1445. https://doi.org/10.1002/hbm.25304

23. Boeckle M, Liegl G, Jank R, Pieh C (2016): Neural correlates of conversion disorder: Overview and meta-analysis of neuroimaging studies on motor conversion disorder. BMC Psychiatry 16: 195. https://doi.org/10.1186/s12888-016-0890-x

24. Drane DL, Fani N, Hallett M, Khalsa SS, Perez DL, Roberts NA (2021): A framework for understanding the pathophysiology of functional neurological disorder. CNS Spectr 26: 555–561. https://doi.org/10.1017/S1092852920001789

25. Weber S, Bühler J, Vanini G, Huber R, Trinka E, Bruckmaier R, et al. (2023): Identification of biopsychological trait markers in functional neurological disorders. Brain 146: 2627–2641. https://doi.org/10.1093/brain/awac442

26. Poldrack RA (2006): Can cognitive processes be inferred from neuroimaging data? *Trends Cogn Sci* 10: 59–63. https://doi.org/10.1016/j.tics.2005.12.004

27. Poldrack RA (2011): Inferring mental states from neuroimaging data: From reverse inference to large-scale decoding. *Neuron* 72: 692–697. https://doi.org/10.1016/j.neuron.2011.11.001

28. Aitchison L, Lengyel M (2017): With or without you: Predictive coding and Bayesian inference in the brain. *Curr Opin Neurobiol* 46: 219–227. https://doi.org/10.1016/j.conb.2017.08.010
